# Effects of Mitochondrial-Derived Peptides (MDPs) on Mitochondrial and Cellular Health in AMD

**DOI:** 10.3390/cells9051102

**Published:** 2020-04-29

**Authors:** Sonali Nashine, M. Cristina Kenney

**Affiliations:** 1 Department of Ophthalmology, Gavin Herbert Eye Institute, University of California Irvine, Irvine, CA 92697, USA; snashine@uci.edu; 2 Department of Pathology and Laboratory Medicine, University of California Irvine, Irvine, CA 92697, USA

**Keywords:** MDPs, mitochondrial-derived peptides, Humanin, HNG, SHLPs, MOTS-c, Age-related Macular Degeneration (AMD), neuroprotection, RPE, mitochondria

## Abstract

Substantive evidence demonstrates the contribution of mitochondrial dysfunction in the etiology and pathogenesis of Age-related Macular Degeneration (AMD). Recently, extensive characterization of Mitochondrial-Derived Peptides (MDPs) has revealed their cytoprotective role in several diseases, including AMD. Here we summarize the varied effects of MDPs on cellular and mitochondrial health, which establish the merit of MDPs as therapeutic targets for AMD. We argue that further research to delve into the mechanisms of action and delivery of MDPs may advance the field of AMD therapy.

## 1. Introduction

In the United States, geographic atrophy in dry AMD (Age-related Macular Degeneration) is a primary cause of vision loss in the elderly [1], and it has limited treatment options compared to those available for wet AMD [2,3]. Among the wide variety of factors that are instrumental in the etiology and pathogenesis of AMD, mitochondrial damage in the Retinal Pigment Epithelium (RPE), leading to RPE dysfunction, contributes significantly. AMD mitochondria are fragmented, have a higher number of lesions, altered ATP synthase activity, as well as compromised protein expression and nuclear-encoded protein import [4,5,6]. Furthermore, as confirmed by ATAC-sequencing, chromatin accessibility is decreased significantly in the RPE in AMD retinas [7].

The human mitochondrial genome is ~16.5 kilobases, circular, double-stranded, and consists of 37 genes that code for 13 proteins of the respiratory chain complexes. These 13 proteins are a part of the electron transport chain and aid in oxidative metabolism and ATP production [8,9]. Some of the critical functions of mitochondria include, but are not limited to, ATP production via respiration, promoting thermogenesis via proton leak, regulation of cellular metabolism and calcium signaling, ROS generation, regulation of apoptosis, ion homeostasis, and heme synthesis [10]. The retina is a part of the central nervous system and is one of the highest energy-demanding tissues in the human body. Glycolysis in the cytosol and mitochondrial oxidative phosphorylation are the two primary sources of energy, i.e., ATP (adenosine triphosphate) generation. Retinal neurons derive their energy mostly from oxidative phosphorylation, which has a substantially higher ATP yield than glycolysis [11]. Unmet retinal energy demand puts the retinal neurons at a high risk of cell death that in turn causes impairment or loss of vision [12]. Mitochondrial function declines with age as a result of accumulated mtDNA damage/mutations [13]. The majority of proteins that support mitochondrial health and function are encoded by the nuclear genome. Therefore, coordinated regulation of mitochondrial and nuclear gene expression is essential to maintain cellular homeostasis [14]. Mammalian mitochondrial retrograde signaling, i.e., transmission of information from the mitochondria to the nucleus, is mediated by G-Protein Pathway Suppressor 2 (GPS2), which also functions as a transcriptional activator of nuclear-encoded mitochondrial genes. GPS2-regulated direct translocation from the mitochondria to the nucleus is essential for the transcriptional activation of a nuclear stress response to mitochondrial depolarization and for supporting basal mitochondrial biogenesis [15].

The mitochondrial genome encodes 22 tRNAs and two ribosomal RNAs, i.e., 12S rRNA and 16S rRNA, both of which are essential in synthesis of mitochondrial proteins. The 12S rRNA gene is 954 base pairs (bp) in length and spans 648–1601 bp of the mtDNA; it occupies 1/16 of the entire mtDNA and has 297 nucleotide substitutions, which account for 31% of the 12S rRNA gene. Furthermore, the 16S rRNA gene is 1559 bp long, spanning 1671–3229 bp of the mtDNA; it occupies 1/10 of the entire mtDNA and has 413 nucleotide substitutions, which account for 25% of the 16S rRNA gene [16]. Both 16S rRNA and 12S rRNA genes carry ORFs (Open Reading Frames) that encode mitochondrial-derived peptides.

## 2. Mitochondrial-Derived Peptides (MDPs)

Novel mitochondrial-derived peptides (MDPs), which are encoded within the mtDNA, serve as signals for organism cytoprotection and energy regulation. The MDPs encoded from the 16S rRNA region of the mtDNA include Humanin and SHLPs, which regulate cell survival and growth via distinct pathways. MOTS-c, encoded from the 12S rRNA region of the mtDNA, plays a key role in regulation of muscle and fat metabolism and prevents hepatic steatosis. Numerous MDPs are well-characterized and are in preclinical development for aging-related diseases [17,18,19,20].

## 3. Humanin

Humanin was the first MDP discovered within the mammalian mitochondrial genome and is encoded from the 16S rRNA coding region of the mtDNA. Humanin cDNA was first discovered in 2001 by functional expression screening of a cDNA library preparation from the occipital cortex of an Alzheimer’s disease patient brain [21]. The study identified clones that protected against neuronal cell death induced by neurotoxic amyloid-β peptides and by mutants of FAD (Familial Alzheimer’s Disease) genes, namely *APP* (Amyloid Precursor Protein), *PS1* (Presenilin 1), and *PS2* (Presenilin 2). A 75 bp open reading frame that codes for a 24-residue peptide was identified and its sequence was found to be similar to that of a human cDNA and 99% identical to the mtDNA sequence; it was therefore named Humanin. Humanin antagonized the cytotoxicity caused by *APP* (Amyloid Precursor Protein (APP), *PS1* (Presenilin 1), and *PS2* (Presenilin 2) mutants, by suppressing amyloid-β_1-42_-triggered neurotoxicity and attenuating neuronal cell death.

### 3.1. Structure

**Figure 1:**
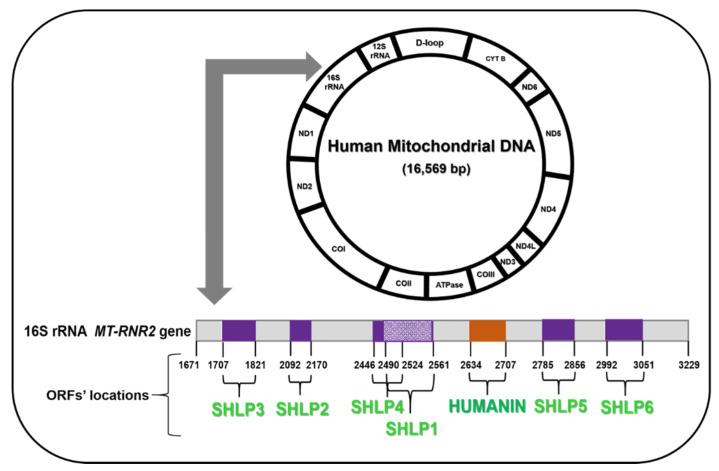
Humanin and Small Human-Like Peptides (SHLPs) Open Reading Frames (ORFs) in the human mitochondrial DNA.

Humanin is a secretory 24-amino acid peptide with the amino acid sequence *H-Met-Ala-Pro-Arg-Gly-Phe-Ser-Cys-Leu-Leu-Leu-Leu-Thr-Ser-Glu-Ile-Asp-Leu-Pro-Val-Lys-Arg-Arg-Ala-OH (H-MAPRGFSCLLLLTSEIDLPVKRRA -OH)* and a molecular weight of 2687.26 Da (Figure 1). Following its discovery, the structure and biological functions of Humanin have been very well characterized. Using a combination of oligonucleotide synthesis, dimerization experiments, and bioinformatics, Yamagishi et al. conducted a comprehensive investigation that led to the identification of the amino acids essential for Humanin secretion and for its neuroprotective function [22]. This study revealed that Humanin is a signal peptide and the core domain of Humanin is formed by Leu9-Leu10-Leu11 residues with Leu10 being the key player. Leu9-Leu10-Leu11 and Pro19-Val20 are absolutely essential for the extracellular secretion of full-length Humanin. The neuroprotective action of Humanin requires Pro19, Ser14, Thr13, Leu12, Leu9, Cys8, Ser7, and Pro3. Ser7 and Leu9 residues are required for the self-dimerization of Humanin, which is necessary for its neuroprotective function. Providing critical insights into the amino acid requirements of Humanin would be useful in synthesis of peptides tailored to perform a specific biological function and for targeted improvement of particular cellular functions.

### 3.2. Tissue Distribution

Measurable levels of Humanin are detectable in plasma, seminal fluid, and cerebrospinal fluid. A circulating Humanin pool has been demonstrated in various tissues of the human body, including hypothalamus, liver, heart, kidney, colon, testes, vasculature, and skeletal muscle [23,24,25,26,27,28]. Circulating plasma levels of Humanin decline with age in both humans and mice [29]. Demonstrating a proportional relationship between Humanin levels and human aging is of particular importance, as this could promote research that aims to boost Humanin levels and thereby delay aging in humans.

### 3.3. Humanin Analogs

Amino acid substitutions in Humanin have led to the development of synthetic Humanin analogs, which are more potent than the endogenous Humanin. Humanin G (HNG) is formed by a Ser to Gly amino acid substitution at position 14. HNGF6A is formed by the Phe to Ala amino acid substitution at position 6 [30].

### 3.4. Humanin Receptors and Regulation

Humanin exerts its cytoprotective effects by binding to receptors intracellularly/extracellularly, regulating the intrinsic mitochondrial pathway, and mediating downstream signaling. Humanin binds to the Formyl-Peptide Receptor-Like Receptor-1 (FPRL-1) and the IL-6 (Interleukin-6) receptor family trimeric receptor complex, comprised of CNTFRα (Ciliary Neurotrophic Factor Receptor α), WSX-1, and GP130 (glycoprotein 130kDa), which are key contributors to the neuroprotective action of Humanin [31,32,33]. Substantial evidence suggests that mitochondrial retrograde signaling is involved in the endocrine regulation of aging and age-related pathologies. IGF-1 (Insulin Growth Factor-1) is a key player in the conserved endocrine pathway, which contributes to lifespan and healthspan. Humanin, a potent mediator of mitochondrial retrograde signaling, is directly regulated by IGF-1, and the levels of Humanin and IGF-1 simultaneously decline with age [34]. Humanin and IGF-1 are known to have distinct yet overlapping functions. By interacting with the C-terminal domain of IGFBP-3 (Insulin-like Growth Factor Binding Protein-3), Humanin interferes with the binding of Importin-β to IGFBP-3, thereby suppressing IGFBP-3-mediated apoptosis [35]. Humanin regulates peripheral insulin action. In this study, peripherally administered HNGF6A in rats conferred insulin sensitivity via hypothalamic STAT3 (Signal transducer and activator of transcription 3) activation [29].

## 4. Humanin Functions

### 4.1. Prevents Apoptosis

Humanin binds to the pro-apoptotic protein BAX and prevents its translocation to the nucleus, thereby antagonizing the apoptotic activity of BAX and inhibiting the release of cytochrome c [36]. This finding reported by Guo et al. in the journal *Nature* was the first thorough study that delineated and reported the interactions of Humanin with the pro-apoptotic BAX protein. Since Humanin is encoded by the mitochondria, this report also suggested a possible retrograde signaling mechanism between the mitochondria and the nucleus that might be contributing to regulation of BAX-mediated apoptosis.

**Figure 2:**
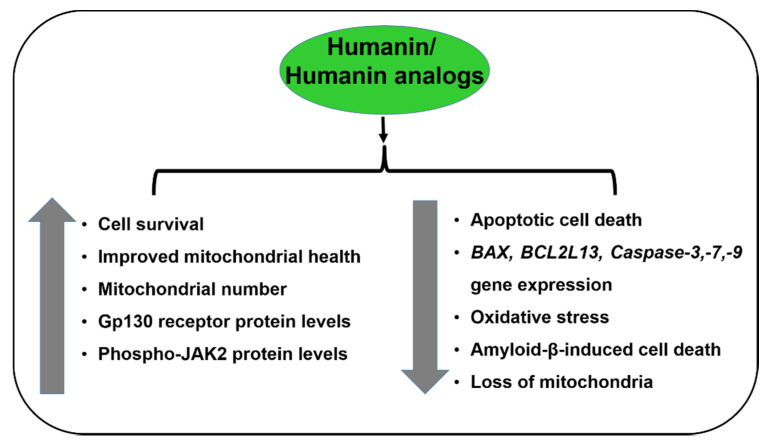
Effects of Humanin/Humanin analogs in RPE/AMD.

Humanin also binds and inactivates BAX-like proteins, such as Bid and BidEL [37]. Exogenously added HNG exerts substantive protective effects in transmitochondrial AMD RPE cells in vitro by (a) rescue of mitochondrial structure and function, (b) inhibiting the action of pro-apoptotic genes/proteins, and (c) intracellular and extracellular humanin receptor modulation (Figure 2) [38].

Humanin is cytoprotective against amyloid-β-mediated toxicity in neuronal cells, both in vitro and in vivo [17,18]; against cerebral ischemia and cardiac damage in mouse models [19,20]; and in numerous neurodegenerative disease models for Alzheimer’s disease, Parkinson’s disease, Huntington’s disease, and Prion diseases [18,19,39,40]. Moreover, Hinton et al. in a comprehensive study published in the highly reputed *IOVS* eye journal demonstrated that Humanin rescues primary RPE cells from oxidative damage and subsequent death in vitro [41]. This showed that Humanin conferred RPE cell protection via two mechanisms: by enhancing mitochondrial biogenesis and function and by activation of STAT3. Further, Humanin mediated suppression of oxidative stress-induced cell senescence and maintained transepithelial resistance in human RPE monolayers. In summary, this study by Hinton et al. suggested the potential of Humanin as a therapeutic candidate for the treatment of geographic atrophy in dry AMD.

Each of the 24 amino acids in the Humanin peptide have a specific function. Serine at position 14 confers neuroprotection [22], but its substitution with glycine generates a variant called Humanin G/ HNG that is 1000-fold more potent than its parent analog Humanin [16]. HNG is known to protect against cell death by preventing mitochondrial dysfunction [26].

Humanin potentially inhibits silver nanoparticles-induced cell death in human neuroblastoma cells by (a) protecting against redox imbalance and oxidative stress-induced DNA damage, (b) increasing mitochondrial membrane potential and enhancing the activity of mitochondrial succinate dehydrogenase, and (c) deactivation of the ER stress pathways that were upregulated by the silver nanoparticles [42].

### 4.2. Prevents Amyloid-β-Induced Toxicity

Amyloid-β is a key component of drusen deposits that are formed in the AMD retinas. Administration of Humanin G reduces amyloid-β loads and inhibits amyloid-β-induced cell apoptosis by (a) restoring amyloid-β-mediated decline in calcium homeostasis, (b) suppressing amyloid-β-induced membrane fluidity changes, (c) restoring mitochondrial membrane potential, and (d) decreasing intracellular reactive oxygen species [43]. This finding is crucial as Humanin G’s ability to mitigate amyloid-β-induced cytotoxicity might be used as a therapeutic approach to delay the progression of dry AMD.

### 4.3. Stress Resistance Against ER Stress-Induced Apoptosis

Humanin exerts its therapeutic benefits by antagonizing the action of a plethora of cellular insults, thereby protecting the RPE cells against cytotoxicity. Treatment with Humanin downregulates the expression of an ER stress marker CHOP (C/EBP Homologous Protein), inhibits ROS (Reactive Oxygen Species) production, and regulates intracellular calcium influx, thereby preventing RPE cell apoptosis [44,45]. Treatment of primary human RPE cells with three ER stress sensors, i.e., Tunicamycin, Brefeldin A, and Thapsigargin, induced mitochondrial damage and dose-dependent loss of RPE cells. However, pretreatment with Humanin provided significant cytoprotection against ER stress-induced cell death by restoring the depleted mitochondrial glutathione (GSH) levels to normal, reducing mitochondrial superoxide generation, and downregulating ER stress-induced apoptotic Caspase 4 and Caspase 3 [46].

### 4.4. Activation of the ERK, AKT, and STAT3 Signaling Pathways

As a secretory peptide, Humanin regulates both intracellular and extracellular signaling pathways. Exogenously administered Humanin both in vitro and in vivo rapidly increases AKT-1 phosphorylation and activates the PI3K (Phosphoinositide 3-Kinase)/AKT pathway; AKT-1 is the AKT Serine/Threonine Kinase-1 protein that plays a role in cell motility, metabolism, and proliferation [47]. Moreover, intraperitoneal injection of Humanin in vivo elevates endothelial NOS (Nitric Oxide Synthase) phosphorylation and also increases the phosphorylation of AMPK (5′ Adenosine Monophosphate-Activated Protein Kinase), a protein which contributes to cellular energy homeostasis [25]. The signaling pathway mediated by Humanin involves its interaction with various molecular entities, including protein kinases, integral membrane receptors, and transcription regulators. In neuronal cell lines, Humanin interacts with and activates the GP130 receptor and mediates its effects via its canonical AKT, ERK1/2 (Extracellular Signal-Regulated Kinase 1/2), and STAT3 signaling cascades. Humanin acts as a primary agonist for the GP130 receptor in neuronal cell lines in vitro, and Humanin treatment transiently activates GP130, thereby mediating cellular protective effects, such as anti-apoptosis, enhanced insulin sensitivity, and protection from hypoxic and ischemic stressors [48].

### 4.5. Preserves Endothelial Function in Atherosclerosis

Oh et al. demonstrated that sustained administration of Humanin G (a more potent analog of Humanin) to ApoE-knockout mice inhibited the progression of atherosclerotic plaques and preserved endothelial function [49].

### 4.6. Prevents Vascular Remodeling and Inflammation

Exogenously added Humanin attenuated angiogenesis, inflammation, apoptosis, and microvascular remodeling in an ApoE-deficient mouse model of atherosclerosis [23].

### 4.7. Cytoprotective Against LDL-Induced Oxidative Stress

Bachar et al. demonstrated that Humanin is expressed in the endothelial cell layer of human blood vessels. In human endothelial cells in vitro, exogenous supplementation of Humanin attenuated oxidized (Ox)-LDL-induced ROS generation and apoptotic cell death by 50% and reduced the levels of cellular ceramide, which is a lipid second messenger that initiates apoptotic signaling cascades. Therefore, Humanin renders protection against Ox-LDL-mediated oxidative stress and is cytoprotective in human vasculature [50].

### 4.8. Protects Germ Cells/Leukocytes—Reduces Cancer Metastases

A recent study by Jia et al. provided evidence of the role of Humanin in maintaining germ cell homeostasis. Substantive Humanin-mediated protection against chemotherapy-induced male germ cell apoptosis both in vivo and ex vivo was observed. The study also demonstrated that this anti-apoptotic effect is primarily mediated via STAT3 and BAX signaling [51].

### 4.9. Germ Cell Apoptosis by Chemo Drugs

Another similar study by Lue et al. demonstrated that Humanin G prevents cyclophosphamide-induced toxicity and death of male germ cells and leukocytes. In this study, HNG protected normal cells against stress and suppressed cancer metastases [52].

### 4.10. Cytoprotection in Carotid Atherosclerotic Plaques

In addition, it has been reported that Humanin is present in carotid atherosclerotic plaques and higher expression of Humanin in symptomatic patients compared to asymptomatic patients could be a part of a stress-response defense mechanism to delay the progression of the disease [53].

## 5. SHLPs (Small Humanin-Like Peptides)

The 16S rRNA region of the mitochondrial DNA also codes for another category of MDPs called Small Humanin-Like Peptides (SHLPs), which include SHLP1, SHLP2, SHLP3, SHLP4, SHLP5, and SHLP6. SHLPs are 24–38 amino acids long and each SHLP may differentially regulate mitochondrial and cellular health and functions. Extensive characterization of SHLP2, SHLP3, and SHLP6 has been detailed in recent literature but the specific biological functions of the SHLPs are still being studied.

### 5.1. SHLP2

**Figure 3:**
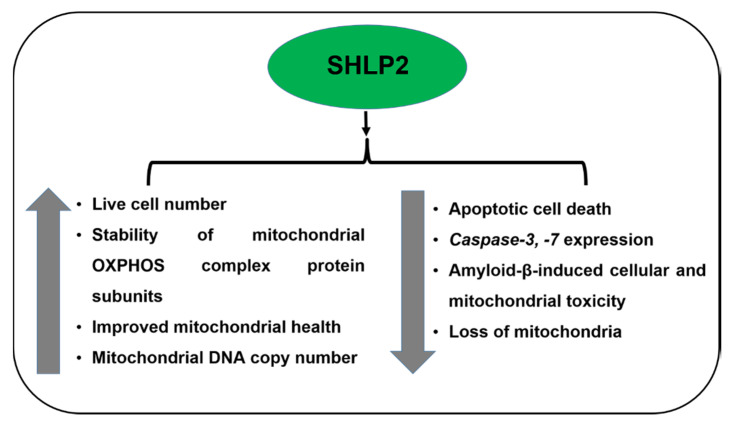
Effects of SHLP2 in AMD.

SHLP2 has a molecular weight of 3017.54 Da and is 26 amino acids long with the sequence *H-MGVKFFTLSTRFFPSVQRAVPLWTNS-OH* (Figure 1). The plasma SHLP2 levels decline with age, suggesting its critical role in aging. SHLP2 stabilizes the AMD mitochondria by preserving the mitochondrial oxidative phosphorylation protein complex subunits (I-V) in AMD RPE transmitochondrial cells, and also promotes mitochondrial metabolism. This study also highlighted the potential of an exogenously added SHLP2 peptide to enhance mitochondria-specific mtGFP fluorescence staining, increase mtDNA copy numbers, and upregulate the *PGC-1α* gene, which is a master regulator of mitochondrial biogenesis [54]. This in vitro study by Nashine et al. is the first study that demonstrates an association between SHLP2 and AMD transmitochondrial RPE cells, and therefore provides a basis for further explorative studies in the field of AMD. Pretreatment with SHLP2 inhibited apoptotic cell death as evidenced by higher live cell numbers and substantive downregulation of effector caspases, namely *Caspase-3* and *Caspase-7*, in AMD RPE cells. Moreover, SHLP2 rescued and protected AMD RPE cybrid cells against amyloid-β-induced toxicity in vitro (Figure 3). Therefore, SHLP2 could be considered a potential therapeutic candidate for macular degeneration. SHLP2 also mediates chaperone-like activity, prevents the misfolding of amyloid polypeptides, and prevents neuronal cell death [55].

### 5.2. SHLP3

SHLP3 is a 38 amino acids-long peptide with the sequence *H-MLGYNFSSFPCGTISIAPGFNFYRLYFIWVNGLAKVVW-OH* and has a molecular weight of 4380.15 Da. SHLP3 increases cellular ATP levels and mitochondrial oxygen consumption rate (OCR) in vitro. SHLP3 suppresses ROS generation, mediates ERK signaling, promotes adipocyte cellular differentiation, and blocks staurosporine-induced apoptosis, thereby promoting mitochondrial and cellular survival and functions. SHLP2 and SHLP3 have insulin-sensitizing effects both in vivo and in vitro, and are known to increase leptin levels. SHLP2 functions as an insulin sensitizer both peripherally and centrally as it enhances peripheral glucose uptake and inhibits glucose production in the liver. SHLP3 also increases IL-6 and MCP-1 levels.

SHLP4 increases cell proliferation in the NIT-1 and 22RV-1 cell lines in vitro. SHLP6 drastically enhances apoptotic cell death in the NIT-1 and 22RV-1 cell lines, and exerts an effect opposite of SHLP2 and SHLP3, which are cytoprotective molecules [56]. Among all the SHLPs, SHLP2 is the only peptide whose cytoprotective function has been established in AMD (Figure 3).

## 6. MOTS-c

**Figure 4:**
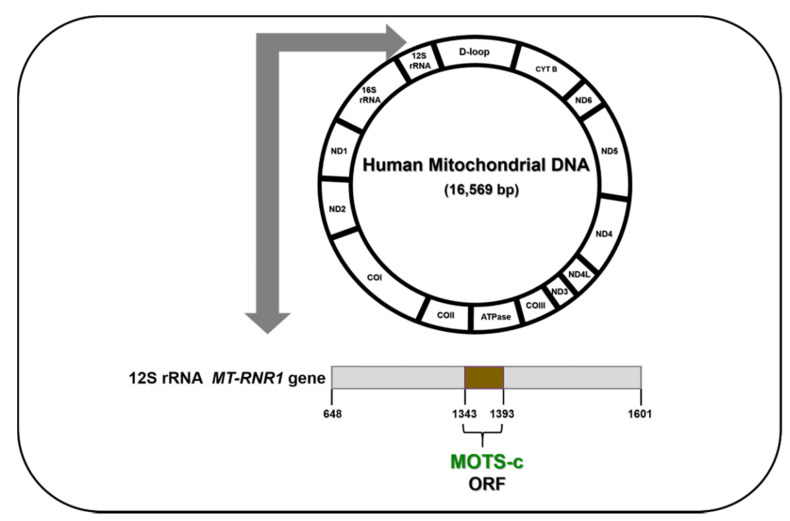
MOTS-c ORF in the human mitochondrial DNA.

**Figure 5:**
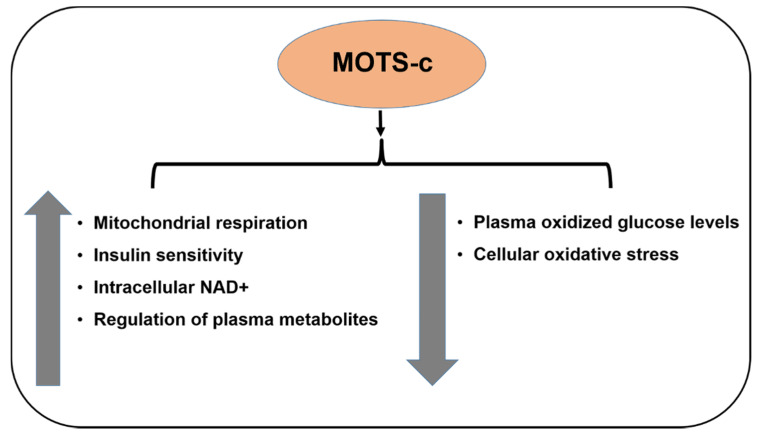
Effects of MOTS-c.

MOTS-c (Mitochondrial ORF (Open Reading Frame) within the Twelve S rRNA c) is encoded in the 12S rRNA region of the mtDNA, and is a 16-amino acid peptide with the sequence *H-MRWQEMGYIFYPRKLR-OH* and a molecular weight 2174.7 Da (Figure 4). It is associated with insulin resistance and is found in plasma, brain, liver, and muscle tissues. MOTS-c enhances insulin sensitivity and regulates plasma metabolites in three metabolic pathways, namely sphingolipid metabolism, monoacylglycerol metabolism, and dicarboxylate metabolism. MOTS-c indirectly decreases cellular oxidative stress by reducing plasma oxidized glucose levels. MOTS-c also activates AKT phosphorylation and AMPK pathways. Moreover, MOTS-c reduces skeletal muscle fatigue and improves performance in vivo [57]. This is the first report by Cohen et al. that delineates the specific roles of MOTS-c and opens new avenues for further research with this MDP. MOTS-c enhances mitochondrial respiration in senescent cells by regulating fatty acid oxidation and increasing the senescence-related effectors [58]. Since MOTS-c levels also decline with age, it has been implicated in the regulation of lifespan and healthspan in organisms. This lifespan/healthspan prolonging ability of MOTS-c may be attributed to (1) MOTS-c-mediated increase in intracellular NAD^+^, a key redox metabolic coenzyme that activates sirtuins, which in turn regulate aging; and (2) MOTS-c-mediated reduction in methionine metabolism via inhibition of the folate/methionine cycle [59]. Although the function of MOTS-c as a crucial player in cell longevity, mitochondrial function, and metabolic homeostasis has been well-established, its specific protective function in the eye is yet to be reported. Since the AMD etiology involves a metabolic component as well as mitochondrial dysregulation, it would be interesting to investigate the cytoprotective effects of MOTS-c in AMD pathology (Figure 5).

CohBar, a clinical-stage biotechnology company focused on the research and development of mitochondria-based therapeutics, has successfully completed a Phase 1a clinical study and initiated the Phase 1b stage of a double-blind, placebo-controlled clinical trial of CB4211 as a potential treatment for nonalcoholic steatohepatitis (NASH) and obesity. CB4211 is the first therapeutic candidate based on a mitochondrial-derived peptide to enter clinical testing in humans. The completed Phase 1a stage of the CB4211 clinical study evaluated safety and tolerability, and the drug was safe and well tolerated after seven days of dosing. The Phase 1b stage of the study will be an assessment of safety, tolerability, and activity in obese subjects with nonalcoholic fatty liver diseases (NAFLD). Assessments will include changes in liver fat assessed by MRI-PDFF, body weight, and biomarkers relevant to NASH and obesity [60].

## 7. Conclusions and Future Directions

The dry and wet forms of AMD have some common denominators in terms of AMD disease pathology, since the characteristic features of dry AMD, i.e., RPE cell apoptosis and accumulation of drusen deposits (in which amyloid-β is a key component), may subsequently lead to choroidal neovascularization observed in wet AMD pathology. To our knowledge, differential effects of MDPs in dry AMD versus wet AMD have not been delineated and reported yet.

Since this manuscript presents the literature that establishes the role of MDPs in mitigating RPE cell apoptosis and reducing amyloid-β-induced toxicity, it suggests that MDPs are potential therapeutic candidates for treatment of dry AMD, and may delay its progression to the late form, i.e., wet AMD. However, the specific therapeutic effects of MDPs in reducing or preventing choroidal neovascularization in wet AMD need to be characterized. Experimentally, the effects of MDPs on the angiogenesis-promoting factors should be tested to evaluate if MDPs are able to downregulate the pro-angiogenic factors and related pathways, and thereby prevent or suppress neovascularization in wet AMD.

In conclusion, MDPs, especially Humanin (and its analogs) and SHLPs, provide cytoprotection in ocular diseases, including AMD, and may be considered as potential therapeutic targets for AMD. However, the use of MDPs as therapeutic agents for AMD will require development of appropriate delivery techniques and formulations. Currently, nanoparticles encapsulating Humanin are being tested in some labs to subsequently facilitate its efficient delivery and sustained action. In a recent study by Li et al., it was demonstrated that the Humanin peptide mediates elastin-like polypeptide nano-assembly and protects human RPE cells against oxidative stress-induced apoptotic cell death. This technology may facilitate cellular delivery of biodegradable nanoparticles with potential protection against AMD [61]. Furthermore, with the development of precision medicine, the use of MDPs can be customized for AMD therapy to match patient needs.
